# Effects of drop height, conveyor belt speed, and acceleration on the welfare of broiler chickens in early and later life

**DOI:** 10.1016/j.psj.2020.08.066

**Published:** 2020-09-15

**Authors:** Mona F. Giersberg, Roos Molenaar, Remco Pieters, William Boyer, T. Bas Rodenburg

**Affiliations:** ∗ Adaptation Physiology Group, Wageningen University & Research, 6700 AH Wageningen, the Netherlands; † Animals in Science and Society, Department Population Health Sciences, Faculty of Veterinary Medicine, Utrecht University, 3508 TD Utrecht, the Netherlands; ‡ Experimental Zoology Group, Wageningen University & Research, 6700 AH Wageningen, the Netherlands; § Ceva Santé Animale, 33501 Liboune, France

**Keywords:** hatchery processing, traumatic injury, radiographic image, fear response, welfare

## Abstract

During automated processing in commercial hatcheries, day-old chicks are subjected to a range of possible mental and physical stressors. Three determinants of the processing line seem to have the potential to affect the birds in particular: drop height from one conveyor belt to another, conveyor belt speed, and acceleration. The aim of this study was to evaluate the effects of these 3 factors on chicken health and welfare in early and later life. In a first trial, chickens were tested on an experimental processing line that was adjusted to different levels of drop heights, belt speeds, and accelerations separately (n = 14 animals per factor and increment). Besides the assessment of several indicators for disorientation during the treatment, postmortem radiographic images were created and analyzed with focus on traumatic injuries. The number of chickens changing their orientation after the drop was affected by drop height (*P* < 0.01), whereas body posture changes were affected both by drop height (*P* < 0.01) and belt speed (*P* < 0.01). Traumatic injuries were found only sporadically and were not related to a certain treatment. In a second trial, chickens that were exposed to a combination of the 3 processing factors were compared with an untreated control group (n = 63 per group) until 15 d of age. There were no differences between the 2 groups regarding BW, welfare scores, and fear-related responses in a novel object and in a tonic immobility test. The present results suggest that the treatments on the experimental conveyor belts affected the birds' health, welfare, and behavior to a limited extend. However, starting at a drop height of 280 mm and a conveyor belt speed of 27 m/min, significantly more chickens were not able to maintain their initial body position on the belt. This indicates that there may be scope for discomfort and welfare impairment if commercial systems are operated with considerably larger drop heights and at higher speeds.

## Introduction

During their first day of life, chickens in commercial hatcheries are subjected to several handling procedures. Processing usually starts at the egg separator, where newly hatched chickens are separated from egg shells and unhatched eggs. The chickens are further transported on a series of conveyor belts until they pass a quality control point, reach a photoelectric counter, and fall into the collection baskets in which they are usually transported to the farms. Depending on type of poultry and intended use, further steps, such as manual sexing, infrared beak trimming, and subcutaneous or spray vaccination, may be included at some point of the processing line. As they move through the system, the chickens are exposed to 3 main determinants of the processing line: conveyor belt speed, drop height, and acceleration from one conveyor to the next. These factors as well as the total length of the processing line can vary considerably among hatcheries, depending on total throughput and level of automation (Knowles et al., 2004).

The potential welfare risks of commercial chick processing are obvious: certain drop heights, speeds, and accelerations may act as physical and mental stressors by causing for instance traumatic injury, discomfort, disorientation, or loss of predictability and controllability. Besides affecting the chicken at the very moment of processing, stressful events during early life can have long-term effects on the development and behavior of the animal later in life (Ericsson et al., 2016). In broiler chickens, thermal stress at an early age resulted in depressed weight gain until 35 d (Altan et al., 2000). Similarly, early transportation and feed deprivation lead to lower BW in chickens up to an age of 21 and 42 d, respectively (Bergoug et al., 2013; de Jong et al., 2017). Furthermore, chickens exposed to a combination of the factors transport and delayed feeding at day-old were more fearful at 30 d of age compared with transported, early-fed chicks (Hollemans et al., 2018). However, previous work focusing on the period and procedures of hatchery processing and their consequences for chicken development and welfare is limited.

Knowles et al. (2004) investigated the technical characteristics of the processing lines in 3 broiler and 3 laying hen hatcheries in the United Kingdom in detail. In addition to total conveyor belt length and speed and height differences of consecutive belts, they measured the cumulative acceleration imposed by each system as well as major events of acceleration within a system. Potential effects on chicken welfare were measured in terms of righting time and body posture at several parts of interest of the processing line and tonic immobility (**TI**) before and after handling. The authors concluded that the processing systems differed considerably in their physical characteristics and thus in relative “roughness” of handling. Chicken welfare seemed to be acceptable in general; however, there was a risk of operating at high velocities and accelerations if systems were not properly setup or maintained. There was a relationship between cumulative acceleration and fearfulness after handling, with chickens exposed to the highest cumulative acceleration acting most fearful in the TI test. Furthermore, higher speed differences between consecutive conveyor belts were associated with higher proportions of birds not being able to maintain a standing body position on the belt. Cumulative mortality rates at 7 d after placement, which were analyzed to assess long-term effects of processing, did not differ between 2 broiler hatcheries with a more and a less “rough” handling system. However, there was no comparison with an unhandled control group. In addition, the well-being of the chickens could be still impaired in ways that were not reflected by increased mortality rates but by more subtle physiological changes.

Hedlund et al. (2019) measured physiological and behavioral stress indicators in laying hen chickens that hatched in a commercial hatchery and went through commercial processing and in an untreated control group that hatched at the research facility. In addition, production performance, plumage damage, and injuries were recorded up to 20 wk of age. In hatchery-treated chicken, baseline corticosterone levels measured after incubation were higher than those of the control chickens that hatched at the research facility. A significant increase in corticosterone levels was observed during commercial processing of the hatchery-hatched birds. These chickens also showed higher corticosterone reactivity in a restraint test, were more fearful, and had higher BW during the first week of life. At a later age, hatchery-processed chickens showed more plumage damage, injuries, increased egg production, and higher estradiol levels. Therefore, it was concluded that hatchery processing was a stressful event that affected the birds both in the short and in the long term. The study by Hedlund et al. (2019) represents a system comparison between commercially processed and untreated chickens because technical characteristics of the processing line, such as height of drops, conveyor belt speed, or acceleration, were not investigated in detail. Thus, it is not clear which determinants of commercial chick processing at which thresholds may contribute to welfare issues at different animal ages.

Therefore, the aim of the present study was to assess the influence of different hatchery processing factors on the welfare of broiler chickens in early and later life under controlled small-scale experimental conditions. In a first trial, the effects of 3 factors (drop height, speed, and acceleration) were tested separately using an experimental processing line. It was hypothesized that with increasing drop height, conveyor belt speed, or acceleration, the chickens would show higher levels of disorientation, more frequent posture changes, and possibly also signs of traumatic injury. In a second trial, broiler chickens were subjected either to a combination of the 3 processing factors on an experimental processing line or a control treatment (no processing) and were followed up for a 2-wk grow-out period. It was expected that the experimental processing treatment would lead to increased fear responses during behavioral tests and might affect further welfare and production indicators.

## Materials and methods

### Animals and Setup Processing Experiment

A total of 360 eggs from a Ross 308 parent stock (flock age: 40 wk) were collected from a commercial hatchery (Lagerwey, Lunteren, The Netherlands) at 18 d of incubation and placed in hatching baskets in a hatcher at the research facility. Both at the hatchery and at the experimental facility, the eggs were subjected to standard incubation procedures. The incubation temperature was maintained at 37.8°C for all eggs. The RH of the incubator was maintained between 50 and 65% throughout incubation. Eggs were turned every 30 min at an angle of 90° and not exposed to light during incubation. When all chickens had hatched, they were weighed, wing sexed, and received a plastic neck tag with an individual number.

An experimental twin-conveyor-belt system was placed in a test room at the research facility. The system consisted of 2 overlapping belts (each approximately 2 m long and 30 cm wide), whose vertical distance to one another, speed, and acceleration could be set manually for the different testing scenarios. After labeling, the chickens were brought from the hatcher room to the test room in small batches. All birds assigned to trial 2 were tested in the morning, birds from trial 1 in the afternoon. To avoid cold stress, the ambient temperature in the test room was maintained at 32°C.

The experiment was approved by the Central Authority for Scientific Procedures on Animals as per Dutch law (no: 1040020186468).

### Trial 1: Effects of Processing Early in Life

#### Processing Treatment

The effects of 3 processing factors were tested separately: drop height (4 increments: 0, 200, 280, and 360 mm at a speed of 14 m/min, no acceleration), speed (4 increments: 0, 14, 20, and 27 m/min at a drop height of 200 mm, no acceleration), and acceleration (3 increments: 0, 0.1, and 0.2 g, with the lower conveyor belt running faster than the upper one, a speed of 14 m/min of the upper conveyor belt and a drop height of 200 mm). Treatments were mixed to avoid confounding between treatment and time of testing. Testing for trial 1 took place between 13:00 and 16:00 h. Chickens were placed in pairs of 1 male and 1 female on the conveyor belt (n = 14 animals per processing factor and increment). The time until the drop and the acceleration varied between conveyor belt speeds, but on average, it was about 3–5 s. Before and after each run, both chickens were subjected to a righting test. For this, the chicken was placed on its back on a table and the time until standing up was measured. During the run on the conveyor belt, orientation (facing forward, backward, sideways) and posture (standing, sitting, lying) were recorded before and after the drop. Finally, chickens were checked for any signs of bruises or injuries. After the trial, all chickens were killed by manual cervical dislocation and frozen at −20°C for more detailed analyses of injuries postmortem.

#### Postmortem Radiographic Images

Before being radiographed, the carcasses (n = 154) were retrieved from the freezer and thawed at ambient temperature. A total of 55 radiographs was generated, with 2 to 3 individual chickens placed in dorsal recumbency on a panel detector (24 × 30 cm). Ventrodorsal radiographic images of the chickens' whole bodies were produced with a stationary radiographic system (Philips SRM0310 X-ray machine, Philips Super CP 80 generator, Philips Optimus M200 gantry, Philips Super CP 50 terminal, Philips XD6028 collimator, Konica Minolta Regius 110HQ digitizer). The working distance was 127 cm. Voltage, load, and exposure time were set to 40 kV, 32 mAs, and 386 ms, respectively.

The radiographs were analyzed as digital images (DCM files) using the software PDI viewer V2.20R03 (Konica Minolta, Inc.). No enhancements, such as grayscale or contrast adjustments, were applied to the radiographs after production. Before detailed evaluation, each image was rotated by 90° clockwise until the chicken's head pointed to the top of the picture and its feed to the bottom. To detect possible traumatic injuries, the long bones of the extremities of each chicken were assessed for fractures, which were further classified by type and localization (proximal or distal) in relation to the respective bone. In addition to fractures, each bone was scanned for splinters/chips (i.e., small pieces of bone or cartilage that broke off from the whole bone) in its immediate proximity, and a distinction was made between proximal and distal bone splinters.

### Trial 2: Effects of Processing Later in Life

For the grow-out experiment, 2 extreme processing treatments were chosen: 0 mm drop height, 0 m/min speed, 0 g acceleration (i.e., no placement on the conveyor belt, control [**C**]) vs. 200 mm drop height, 27 m/min speed, and 0.2 g acceleration (treatment [**T**]). At day 1, the same measurements were taken as in trial 1. Control chicks were not placed on the conveyor belt system but were subjected to the same handling procedures and the righting test, allowing approximately the same time interval between the 2 righting tests for both T and C chicks. The birds for trial 2 were tested between 9:00 and 12:00 h. Treatments were mixed to avoid effects of time of testing. After processing, the chickens were kept in groups of 9 (4–5 birds of each sex) in 7 floor pens (200 × 100 × 50 cm) per treatment. Pens provided wood shavings on the floor, a drinking line with 4 nipples, and a feeder. A novel object (**NO**) test was carried out at day 1, 8, and 15. The NO used was a plastic frog with a height of approximately 10 cm and a width of approximately 5 cm. The observer placed the NO on the floor in the middle of the pen and recorded for 2 min every 10 s the number of chickens within 25-cm radius of the NO. At day 8 and 15, 28 chickens per treatment (= 2 male and 2 female chickens per pen) were subjected to a TI test. Birds were selected randomly, and repeated testing of the same bird was avoided. The TI was conducted in the same compartment where the animal pens were located as well, on a table in the hallway in front of the pens. Each bird was placed on its back in a metal cradle and restrained in this position for 10 s. If the chickens stayed immobile for at least 10 s after releasing, time monitoring was continued until the bird righted itself or until a maximum TI duration of 5 min was reached. If the chicken returned to an upright position within 10 s, TI was induced again up to a maximum of 5 inductions. The experimenter recorded the number of successful TI inductions, the number of TI induction attempts needed, TI duration, and vocalization (yes/no) during TI. The NO was conducted first, followed by the TI test. Subsequently, all tested chickens were weighed and scored for plumage cleanliness, hock burn, and footpad dermatitis (in accordance with Welfare Quality, 2009). After data collection on day 15, all chickens were killed humanely.

### Statistical Analyses

All statistical analyses were performed using the software SPSS Statistics (version 25; IBM, Armonk, NY). Data were visually examined for normal distribution by creating histograms including the Gaussian distribution curve and homoscedasticity was tested as per the Levene procedure. Depending on distribution, data structure, and variable characteristics, data were subjected to the procedures described as follows. Trial 1: The target variable “righting time” was analyzed for within-subjects effects (righting time of an individual chick before vs. after treatment) and for between-subjects effects (height, speed, and acceleration) by means of repeated measures ANOVAs. GLM models with a multinomial distribution and a logit link function were applied to test for processing effects (height, speed, and acceleration) on orientation and posture before the drop. Binary variables were created for “orientation change” and “posture change,” which indicated whether a chicken had any other orientation or posture after the drop. These variables were analyzed for processing effects by means of GLM models with a binomial distribution and a logit link function. Trial 2: A repeated measures ANOVA was used to analyze the effect of processing on righting time. GLM models were used to analyze the remaining response variables of trial 2. A normal distribution was applied for BW, supplemented with a log link function for birds approaching the NO and TI durations. Models with a binomial distribution and a logit link function were used for plumage cleanliness, successful TI inductions, and vocalization during TI. A Poisson distribution with a log link function was applied for number of TI induction attempts. Models included the fixed effects of treatment and d and the random effect of pen within treatment. Multiple comparisons were adjusted by Bonferroni correction. Differences between the tested parameters were considered to be significant if *P*-values were <0.05. All data are presented as mean ± SEM.

## Results

### Trial 1: Effects of Processing Early in Life

#### Processing Treatment

The average time birds took to erect themselves was <4 s in all treatment groups (Table 1). Regarding the processing factor height, the righting times before and after processing did not differ (F_1,52_ = 1.63, *P* = 0.21). Similar results were obtained for the factors speed (F_1,52_ = 0.81, *P* = 0.37) and acceleration (F_1,39_ = 1.04, *P* = 0.31). Within the treatment group, the righting times did not differ among the tested increments (height: F_3,52_ = 1.66, *P* = 0.19; speed: F_3,52_ = 0.27, *P* = 0.85; acceleration: F_2,39_ = 0.84, *P* = 0.44).

**Table 1:** Mean righting times and numbers of orientation and posture changes of day-old chickens that were subjected to different processing treatments (height, speed, acceleration).

Treatment		n	Mean righting time (s ± SEM)		Birds with orientation change (#)	Birds with posture change (#)
Processing factor	Increment		Before processing	After processing		
Height difference (mm)	0	14	1.07 ± 0.07	1.00 ± 0.00	-	-
	200	14	1.07 ± 0.07	1.14 ± 0.10	3	2
	280	14	1.00 ± 0.00	1.21 ± 0.43	8	14
	360	14	1.36 ± 0.23	1.43 ± 0.25	11	12
Speed (m/min)	0	14	1.07 ± 0.07	1.07 ± 0.07	-	-
	14	14	1.21 ± 0.16	1.00 ± 0.00	7	4
	20	14	1.14 ± 0.10	1.14 ± 0.10	9	8
	27	14	1.07 ± 0.07	1.07 ± 0.07	8	13
Acceleration (g)	0	14	3.71 ± 2.56	1.07 ± 0.07	9	4
	0.1	14	1.36 ± 0.23	1.07 ± 0.07	9	9
	0.2	14	1.07 ± 0.07	1.36 ± 0.23	4	9

At the beginning of the experiment, there was no difference in the orientation on the conveyor belt (facing forward, backward, or sideways) among chickens from the different treatment groups (height: F_6,48_ = 0.87, *P* = 0.53; speed: F_4,36_ = 0.44, *P* = 0.78; acceleration: F_4,36_ = 1.01, *P* = 0.42). An effect of processing on orientation change after the drop from the first conveyor belt was only observed in the height treatment, with more chickens changing their orientation at a drop height of 360 mm compared with 200 mm (Table 1; F_2,39_ = 7.06, *P* < 0.01).

During all trials, no chicken was observed in a lying position, neither before nor after the drop on the second conveyor belt. Similar to orientation, the body positions of the chickens within each treatment did not differ at the beginning of the run (height: F_3,52_ = 0.64, *P* = 0.60; speed: F_2,39_ = 0.18, *P* = 0.84; acceleration: F_2,39_ = 0.52, *P* = 0.60), and about 90% of the birds were standing on the conveyor belt. In the height treatment group, higher proportions of birds had changed their posture to “sitting” after a drop of 280 and 300 mm compared with a drop height of 200 mm (F_2,39_ = 31.99, *P* < 0.01). Similar effects were found in the speed treatment with more posture changes at a speed of 27 m/min compared with 14 and 20 m/min (F_2,39_ = 11.12, *P* < 0.01). In contrast, acceleration had no effect on posture change after the drop (F_2,39_ = 2.22, *P* = 0.12).

After processing, no bruises or other visible injuries were detected in chickens from any treatment group.

#### Detection of Traumatic Injuries by Radiographic Image Analyses

Details on the number of assessed bones and diagnostic findings in the radiographs of chickens subjected to the different processing treatments of trial 1 are presented in Supplementary Table 1 (Supplementary Material). In general, all produced images and all displayed chickens were evaluable. However, the placement of carcasses on the panel detector resulted in overlying tissues which made it impossible to assess 3% of the total number of 3,080 examined bones. Except for 2 fractured tibiae (1 chicken from the treatment drop height 200 mm and 1 from the treatment speed 0 m/min), all bones were rated as “no abnormality detected.” Bone splinters/chips were not observed.

### Trial 2: Effects of Processing Later in Life

As in trial 1, the average righting times on day 1 were <4 s and did not differ before and after processing (F_1,133_ = 1.03, *P* = 0.31) or between the T and C groups (F_1,133_ = 1.37, *P* = 0.24). Because C chickens were not placed on the experimental conveyor belt, orientation and posture change could not be assessed in this group. A change of orientation and posture after the drop was observed in 41.6 and 79.5% of the T chickens, respectively. Again, no apparent bruises or injuries were observed visually.

There was no mortality throughout the entire grow-out period. Control and T chickens grew from 173.19 ± 2.59 g and 176.29 ± 1.99 g at day 8 to 472.56 ± 7.66 g and 483.97 ± 6.40 g at day 15, respectively. BW was affected by age (F_1,249_ = 3731.64, *P* < 0.01), with older chickens being heavier but not by treatment (F_1,249_ = 0.63, *P* = 0.43). Similarly, plumage cleanliness did not differ between the C and T groups (F_1,249_ = 1.22, *P* = 0.27). At both assessment days, > 50% of all birds had clean plumage, the rest was scored 1 (i.e., feathers/skin in the vent area slightly dirty). There was only 1 chicken (T group, day 15) with a footpad score of 1 (i.e., small superficial lesions, slight discoloration on a limited area, mild hyperkeratosis), and no animals with hock burns.

**Figure 1:**
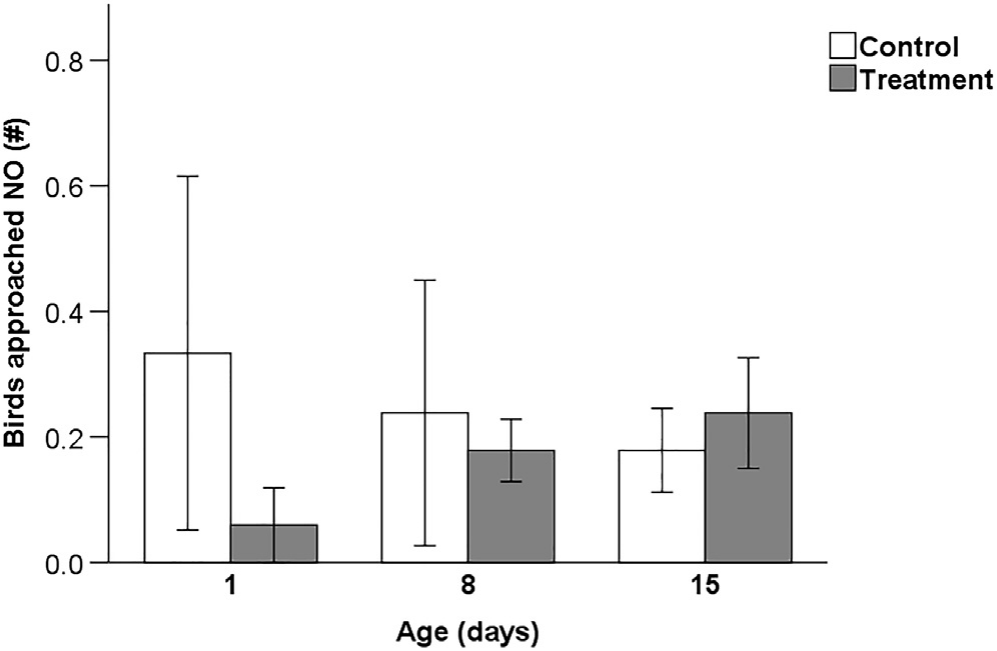
Mean number (±SEM) of chickens approaching the novel object (NO) in a NO test at 1, 8, and 15 d of age for chickens of control pens (control, n = 7) and chickens of pens subjected to processing at day-old (treatment, n = 7).

The mean numbers of chickens approaching the NO within 2 min testing time at day 1, 8, and 15 are shown in Figure 1. No differences were found between treatments (F_1,38_ = 0.71, *P* = 0.41) and days (F_1,38_ = 1.75, *P* = 0.19).

**Figure 2:**
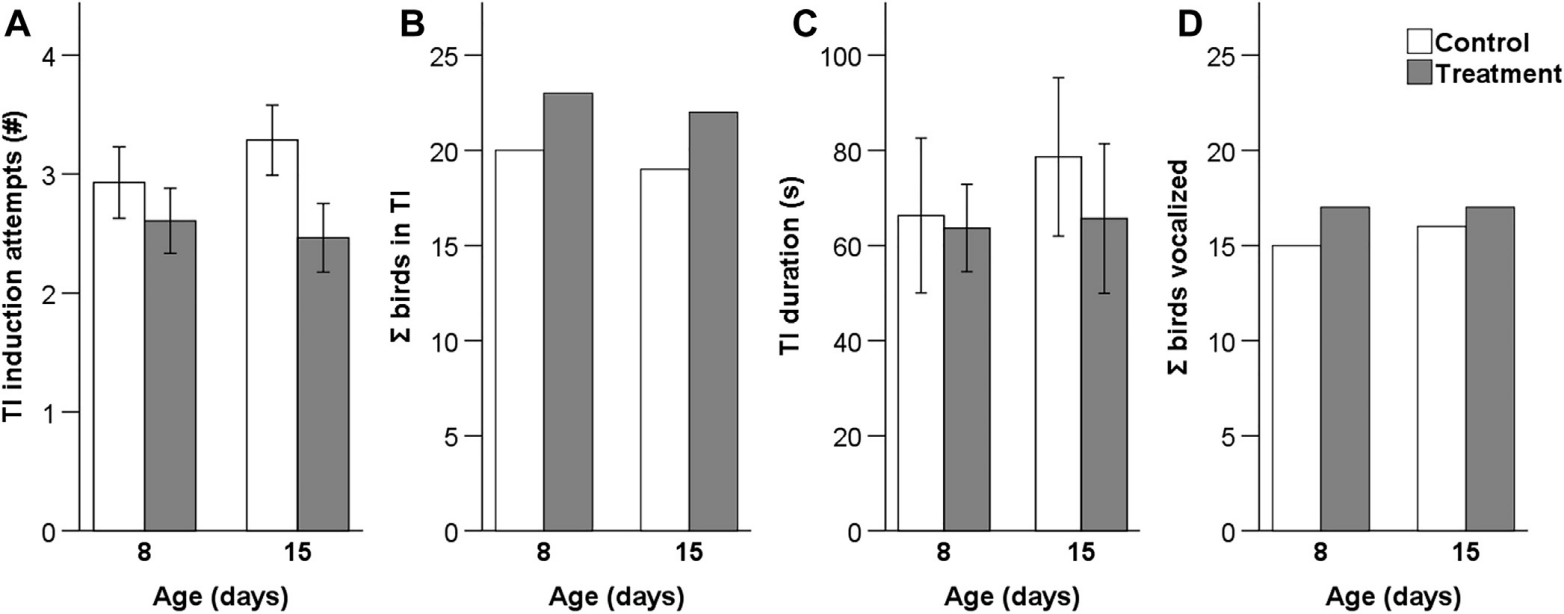
Responses in a tonic immobility (TI) test at 8 and 15 d of age of control chickens (control) and chickens subjected to processing at day-old (treatment): (A) Mean number (±SEM) of TI induction attempts (control, n = 28; treatment, n = 28 per d), (B) sum of birds with effective TI, (C) mean TI durations (±SEM), and (D) sum of birds vocalizing. N in (C,D) refers to birds with effective TI (shown in B), d8: control, n = 20; treatment, n = 23; d15: control, n = 19; treatment, n = 22.

In the TI test, an average of 2 to 3 attempts was needed to induce TI in both groups and at all ages (Figure 2). There was no difference in the number of induction attempts between groups (F_1,109_ = 2.46, *P* = 0.12) and day (F_1,109_ = 0.11, *P* = 0.74). Similarly, the number of chickens win which TI could be induced effectively (<5 attempts) did not differ between treatment groups (F_1,109_ = 0.63, *P* = 0.43) and day (F_1,109_ = 0.21, *P* = 0.65; Figure 2). Mean TI durations were <80 s in both groups and at all ages (Figure 2). Tonic immobility durations were not affected by the treatment group (F_1,89_ = 0.30, *P* = 0.58) and day (F_1,89_ = 0.24, *P* = 0.62). At 15 d, C chickens seemed to remain longer in TI than T chickens. However, pairwise comparisons showed that this difference was not significant (F_1,89_ = 2.82, *P* = 0.09). There was no significant effect of processing treatment (F_1,89_ = 0.20, *P* = 0.66) and day (F_1,89_ = 0.67, *P* = 0.42) on vocalization (Figure 2).

## Discussion

The objective of the present investigation was to evaluate the effects of the 3 processing factors drop height, conveyor belt speed, and acceleration on broiler chicken welfare at the moment of handling and later in life. In general, the treatments on the experimental conveyor belts seemed to affect the birds' health, welfare, and behavior to a limited extend. However, it was shown that starting at a drop height of 280 mm and a conveyor belt speed of 27 m/min, significantly fewer chickens regained a standing body position on the belt after the drop.

The 3 processing factors tested separately in trial 1 mainly affected the orientation and body posture of the chickens after the drop from the first onto the second conveyor belt. Particularly, a higher number of chickens showed a change in orientation, that is facing forward, backward, or sideways at a height difference of 360 mm between the consecutive conveyor belts. Both drop height and conveyor belt speed affected the number of chickens regaining a standing body position after the drop, with drop heights of greater than 280 mm and speeds of 27 m/min resulting in fewer birds standing. Similar results were obtained by Knowles et al. (2004) who observed that in 4 of 6 commercial hatcheries, 100% of the chickens did not regain a standing position at specific transition points with maximum height differences of 350 to 550 mm and largely varying speed differences and cumulative accelerations. Chickens may prefer to quickly regain a standing position after a drop, as the controllability of a situation has been associated with reduced stress responses, even though the animals might not be able to avoid the aversive stimuli or stressor completely (Bassett and Buchanan-Smith, 2007). Nevertheless, the righting test as a further measure of the degree of disorientation and discoordination did not reveal any differences between the times the chickens took to erect themselves before and after the run on the experimental conveyor belt or among the different processing treatments. In contrast, Knowles et al. (2004) found that the longest righting times coincided with those parts of the processing systems that appeared to expose the birds to the “roughest” handling procedures. However, total conveyor belt lengths, cumulative accelerations, and in some hatcheries also maximum drop height and speed differences were larger than those of the present experimental setup. In addition, in the present study, the birds ran on the conveyor belts in pairs, whereas under commercial conditions chickens are usually processed at higher densities. Therefore, future research should examine whether it would be feasible to operate commercial processing lines at maximum drop heights of 200 mm, speeds of 20 m/min, and accelerations of 0.2 g and thus lower than the thresholds that were found to affect the chickens' behavior during handling.

There was no radiographic evidence of traumatic injuries in day-old chickens from trial 1, except for 2 chickens with a fractured shank bone. One of the chickens was subjected to a drop height of 200 mm, whereas the other one was from the control group of the speed treatment, which experienced no change in conveyor belt speed. Thus, fractures occurred with an overall very low incidence (<2% of all animals affected), and, in addition, not in the groups with the most severe processing treatments. Therefore, they can be considered as incidental findings that are unlikely to be related to a specific treatment. Research on early bone development in chickens and bone composition after hatch is scarce. However, the low incidence of fractures during processing may be explained by the characteristic bone structure of young chickens. During the first week of live, bone density and percentage ash increase sharply, suggesting that bone maturation by replacing water by mineralized osteoid takes place rapidly after hatch (Wise, 1970). Therefore, at day-old, the larger proportion of organic material in the chickens' bones may result in a higher flexibility and decreased susceptibility to fractures. Similar to the findings of the righting test, the lack of traumatic injuries might also be attributed to the experimental setup with a shorter conveyor belt and a lower animal density compared with commercial hatchery conditions. Knowles et al. (2004) reported that under commercial conditions, a number of chickens escaped the processing system and fell to the floor. For future research in commercial hatcheries, it would be worthwhile to quantify these chickens and to examine them for traumatic injuries. In addition, it should be kept in mind that processing may cause more subtle injuries, such as small hematomas or bruises in deeper tissues, which cannot be detected by visual scoring or in radiographic images but may be painful for the chicken.

Similarly, hatchery handling may lead to smaller physiological changes that are not reflected in behavioral tests. Hedlund et al. (2019) measured significantly higher corticosterone levels in in hatchery-treated layer chickens than in control birds from the same batch of eggs that hatched at the research facility. However, the hatchery treated birds had already higher corticosterone levels after they were taken from the hatcher, that is, before the run on the processing line, which suggests that additional factors, for instance the noise level of the commercial hatcher, influenced the chickens' stress responses. Until 41 d of age, these chickens had a higher corticosterone reactivity in a restraint test than the control birds (Hedlund et al., 2019). These long-term effects were not observed in a TI test, which measures fear-related behaviors (Forkman et al., 2007). This is in line with the findings of the present investigations: in trial 2, the responses measured in an NO and in a TI test did not differ between birds treated on the conveyor belt and the control group, suggesting that experimental processing had no effects on fearfulness. Similarly, there was no difference in vocalization during TI between processed and unhandled chickens. Vocalizing during social isolation is seen as a risky behavior allowing the bird to regain contact with its social group (Marx et al., 2001). Therefore, chickens vocalizing less during social isolation might be considered as more fearful.

Regarding production, Knowles et al. (2004) found no effects of processing on first-week mortality, whereas Hedlund et al. (2019) observed that hatchery-processed layer chickens had higher BW throughout the study and laid more and larger eggs after reaching maturity. In contrast, broiler chickens from the treatment and the control groups in trial 2 of the present study showed no mortality at all and no difference in BW up to 15 d of age. Again, this may be due to the relatively mild experimental treatment, with particularly shorter conveyor belt length, lower cumulative acceleration, lower noise levels, and shorter total handling time compared with commercial hatchery processing (Knowles et al., 2004). Furthermore, the chickens in the present study did not hatch in a commercial hatchery and were not subjected to transport at day-old, although both factors could add to the effects of processing in a commercial context.

It should be noticed that the present study focused on the effects of processing line factors and thus on handling and treatment procedures after hatch. However, besides the posthatching treatment, the conditions during the first 18 d of incubation in the commercial hatchery, for instance noise levels in the hatcher, the transport of eggs to the research facility at day 18 of incubation, and the associated change of prehatching environments, might influence the chickens' responses after hatch. Although prehatching treatments were kept the same for all chickens in the present study, their effects on the birds might have overruled potentially slighter effects of the posthatching procedures on the experimental conveyor belt. In future studies, it would therefore be interesting to include an additional control group that is incubated under the controlled conditions of a research facility during the whole incubation period.

Processing on an experimental conveyor belt testing the factors drop height, conveyor belt speed, and acceleration had limited effects on chicken health, behavior, and welfare in early and later life. Automated handling mainly affected the chickens' ability to maintain a stable body posture when the processing line was operated at a drop height of 280 mm and a conveyor belt speed of 27 m/min. However, caution must be paid when applying the present results to commercial hatchery procedures. Under commercial conditions, chickens may be exposed to the studied processing factors in a combination, at more severe levels, and for a longer time. Therefore, it should be further investigated which drop heights, conveyor belt speeds, and accelerations are common in practice, which effect these determinants have on the birds, and whether it is feasible to run commercial processing lines lower than the critical thresholds observed in the present study.
