# Supplementary material for: Effects of drop height, conveyor belt speed, and acceleration on the welfare of broiler chickens in early and later life
Source: Poult Sci. 2020 Sep 15;99(12):6293–9. doi: 10.1016/j.psj.2020.08.066 (PMC7705003; doi:10.1016/j.psj.2020.08.066)
Supplement: Supplementary Table 1 [file mmc1.docx]

**Table S1.** Number of evaluated bones and overview of diagnostic findings in post mortem radiographic images of day-old chickens from 3 different processing treatments.

|  |  | Treatment | | | | | | | | | | |
| --- | --- | --- | --- | --- | --- | --- | --- | --- | --- | --- | --- | --- |
|  |  | Height difference (mm)  n = 14 chickens/increment | | | | Speed (m/min)  n = 14 chickens/increment | | | | Acceleration (g)  n = 14 chickens/increment | | |
| Bones |  | 0 | 200 | 280 | 360 | 0 | 14 | 20 | 27 | 0 | 0.1 | 0.2 |
| Humerus | n | 28 | 28 | 28 | 28 | 28 | 28 | 28 | 28 | 28 | 28 | 28 |
|  | n.a. | 7 | 4 | 2 | 5 | 0 | 2 | 2 | 3 | 1 | 1 | 3 |
|  | NAD | 21 | 24 | 26 | 23 | 28 | 26 | 26 | 25 | 27 | 27 | 25 |
|  | Dx | - | - | - | - | - | - | - | - | - | - | - |
|  |  |  |  |  |  |  |  |  |  |  |  |  |
| Ulna | n | 28 | 28 | 28 | 28 | 28 | 28 | 28 | 28 | 28 | 28 | 28 |
|  | n.a. | 2 | 2 | 0 | 1 | 0 | 2 | 0 | 2 | 0 | 0 | 2 |
|  | NAD | 26 | 26 | 28 | 27 | 28 | 26 | 28 | 26 | 28 | 28 | 26 |
|  | Dx | - | - | - | - | - | - | - | - | - | - | - |
|  |  |  |  |  |  |  |  |  |  |  |  |  |
| Radius | n | 28 | 28 | 28 | 28 | 28 | 28 | 28 | 28 | 28 | 28 | 28 |
|  | n.a. | 2 | 2 | 0 | 1 | 0 | 2 | 0 | 2 | 0 | 0 | 2 |
|  | NAD | 26 | 26 | 28 | 27 | 28 | 26 | 28 | 26 | 28 | 28 | 26 |
|  | Dx | - | - | - | - | - | - | - | - | - | - | - |
|  |  |  |  |  |  |  |  |  |  |  |  |  |
| Meta-carpus | n | 28 | 28 | 28 | 28 | 28 | 28 | 28 | 28 | 28 | 28 | 28 |
|  | n.a. | 1 | 1 | 0 | 1 | 0 | 2 | 0 | 2 | 1 | 0 | 2 |
|  | NAD | 27 | 27 | 28 | 27 | 28 | 26 | 28 | 26 | 27 | 28 | 26 |
|  | Dx | - | - | - | - | - | - | - | - | - | - | - |
|  |  |  |  |  |  |  |  |  |  |  |  |  |
| Phalanges prox. | n | 28 | 28 | 28 | 28 | 28 | 28 | 28 | 28 | 28 | 28 | 28 |
|  | n.a. | 0 | 1 | 0 | 1 | 0 | 4 | 0 | 3 | 2 | 0 | 2 |
|  | NAD | 28 | 27 | 28 | 27 | 28 | 24 | 28 | 25 | 26 | 28 | 26 |
|  | Dx | - | - | - | - | - | - | - | - | - | - | - |
|  |  |  |  |  |  |  |  |  |  |  |  |  |
| Femur | n | 28 | 28 | 28 | 28 | 28 | 28 | 28 | 28 | 28 | 28 | 28 |
|  | n.a. | 1 | 1 | 2 | 2 | 2 | 3 | 1 | 1 | 4 | 2 | 0 |
|  | NAD | 27 | 27 | 26 | 26 | 26 | 25 | 27 | 27 | 24 | 26 | 28 |
|  | Dx | - | - | - | - | - | - | - | - | - | - | - |
|  |  |  |  |  |  |  |  |  |  |  |  |  |
| Tibia | n | 28 | 28 | 28 | 28 | 28 | 28 | 28 | 28 | 28 | 28 | 28 |
|  | n.a. | 0 | 0 | 0 | 0 | 0 | 0 | 0 | 0 | 0 | 1 | 0 |
|  | NAD | 28 | 27 | 28 | 28 | 27 | 28 | 28 | 28 | 28 | 27 | 28 |
|  | Dx | - | 1^a^ | - | - | 1^b^ | - | - | - | - | - | - |
|  |  |  |  |  |  |  |  |  |  |  |  |  |
| Fibula | n | 28 | 28 | 28 | 28 | 28 | 28 | 28 | 28 | 28 | 28 | 28 |
|  | n.a. | 0 | 0 | 0 | 0 | 0 | 0 | 0 | 0 | 0 | 1 | 0 |
|  | NAD | 28 | 28 | 28 | 28 | 28 | 28 | 28 | 28 | 28 | 27 | 28 |
|  | Dx | - | - | - | - | - | - | - | - | - | - | - |
|  |  |  |  |  |  |  |  |  |  |  |  |  |
| Metatarsus | n | 28 | 28 | 28 | 28 | 28 | 28 | 28 | 28 | 28 | 28 | 28 |
|  | n.a. | 2 | 0 | 0 | 0 | 0 | 0 | 0 | 0 | 0 | 0 | 1 |
|  | NAD | 26 | 28 | 28 | 28 | 28 | 28 | 28 | 28 | 28 | 28 | 27 |
|  | Dx | - | - | - | - | - | - | - | - | - | - | - |
|  |  |  |  |  |  |  |  |  |  |  |  |  |
| Phalanges dist. | n | 28 | 28 | 28 | 28 | 28 | 28 | 28 | 28 | 28 | 28 | 28 |
|  | n.a. | 1 | 0 | 0 | 0 | 0 | 0 | 0 | 0 | 0 | 0 | 1 |
|  | NAD | 27 | 28 | 28 | 28 | 28 | 28 | 28 | 28 | 28 | 28 | 27 |
|  | Dx | - | - | - | - | - | - | - | - | - | - | - |

n = total number of evaluated bones or groups of bones (phalanges); n.a. = not assessable bones; NAD = bones with no abnormality detected; Dx = diagnosis, ^a^ = fracture, oblique displaced, distal, ^b^ = fracture, oblique non-displaced, distal.
